# Comparative Analysis of Two-Lead DX-Based CRT Versus Conventional Three-Lead CRT-D: Results from a Single-Center Prospective Study

**DOI:** 10.3390/jcm14248746

**Published:** 2025-12-10

**Authors:** Alessandro Carecci, Mauro Biffi, Mirco Lazzeri, Andrea Quaranta, Lorenzo Bartoli, Alberto Spadotto, Cristian Martignani, Andrea Angeletti, Igor Diemberger, Giulia Massaro, Matteo Ziacchi

**Affiliations:** 1 Department of Medical and Surgical Sciences, Institute of Cardiology, University of Bologna, Via Massarenti 9, 40138 Bologna, Italy; 2 Institute of Cardiology, IRCCS Azienda Ospedaliero-Universitaria di Bologna, Via Massarenti 9, 40138 Bologna, Italy; 3 Cardiovascular Department, San Donato Hospital, 52100 Arezzo, Italy

**Keywords:** cardiac resynchronization therapy, heart failure, atrioventricular synchrony, sick sinus syndrome, leads loss of function

## Abstract

**Background/Objectives**: Cardiac resynchronization therapy with defibrillator (CRT-D) is a well-established therapy for patients with heart failure (HF) and intraventricular conduction delays, but a non-negligible risk of infection and of lead functionality loss overtime is related to intravascular hardware. The novel DX system enables atrial sensing through a floating dipole integrated into the ICD lead, reducing the intravascular burden. In this prospective non-randomized study, we aimed to evaluate the safety and efficacy of a two-lead DX-based CRT system compared to a conventional three-lead (3L) CRT-D system. **Methods**: A total of 210 patients meeting CRT indications and no signs of sick sinus syndrome (SSS) (baseline HR ≥ 45 bpm, or at least 85 bpm at 6 min walking test) were enrolled. Patients were assigned to either the CRT-DX or conventional 3L CRT-D group. The primary endpoint was a composite clinical response, defined as the freedom from cardiovascular death, HF hospitalization, or new-onset atrial fibrillation (AF). **Results**: After a mean follow-up of 46.5 ± 1.9 months, both groups had comparable clinical and instrumental outcomes. CRT-DX patients exhibited higher atrial sensing amplitudes and no significant differences in loss of lead function. **Conclusions:** In conclusion, the CRT-DX system provides equivalent clinical and echocardiographic benefits compared to conventional CRT-D in patients without an indication for atrial pacing. This supports the use of the DX system as a safe and effective alternative in the majority of CRT recipients.

## 1. Introduction

Cardiac resynchronization therapy (CRT) represents one of the major advancements in the treatment of heart failure (HF) with reduced ejection fraction (HFrEF) in recent years [1]. In fact, in patients with intraventricular electro-mechanical dyssynchrony (mainly with left bundle branch block -LBBB- and wide QRS > 150 ms) and reduced ejection fraction (≤35%), CRT has unequivocally demonstrated its ability to promote favorable left ventricular (LV) remodeling, decreased HF hospitalizations, mortality, and improved quality of life [2,3,4,5,6]. Despite its benefits, CRT implantation carries a considerable risk of complications, particularly those related to the presence of intravascular hardware. In a retrospective study of over 20,500 implanted devices (ICD or CRT-D), a mechanical complication rate (requiring catheter revision) at 5 years was observed to be approximately 10% (10.7% for CRT and 8% for ICD), rising to 25% as the follow-up was extended to 10 years. The infection rate at 5 years was 2.9% for ICDs and 3.9% for CRT-Ds [7]. Similar results have been confirmed in other patient cohorts, with risks increasing as the number of procedures (battery replacement, revision, upgrade) increases [8]. Factors associated with a higher risk of developing mechanical complications both in the short and long term included female sex, CRT-D, and younger age at implantation; meanwhile, male sex, older age, diabetes mellitus, kidney disease, atrial fibrillation (AF), and HF were more strongly associated with the risk of infectious complications [9].

In recent years, the introduction of the DX lead, a 7.8 French single-coil active-fixation ICD bipolar lead provided with a wide atrial dipole capable of atrial sensing, has enabled CRT delivery by only two leads (one DX lead and one coronary sinus lead), while maintaining adequate atrioventricular synchrony [10,11].

The atrial dipole is provided by two electrodes, spaced 15 mm apart and positioned 15–17 cm proximally from the catheter tip, ensuring atrial sensing and diagnostics. The recorded signal is amplified four-fold and then filtered with a band-pass filter to allow the detection of the P-wave while excluding frequencies outside the atrial signal range.

It has been demonstrated that the DX system provides equivalent capability in detecting atrial activity and discriminating supraventricular tachyarrhythmias (SVT) compared to ICDs with a dedicated atrial lead [12,13,14,15]. This system can therefore be considered in patients who do not require atrial pacing, including those with AF defined as “permanent” based on surface electrocardiography, considering that around 13% of these patients have proven to spontaneously revert to sinus rhythm during follow-up [16].

The potential advantages of using a CRT-D with DX system (CRT-DX) include a possible reduction in procedural times and a decrease in complications related to catheter use, both those specific to the atrial catheter and those caused by the additional burden of intravascular hardware. In 2017, our group published a first comparative study between CRT-DX and CRT-D in a cohort of 37 patients, showing that the CRT-DX system can provide adequate resynchronization therapy at an average 3 year follow-up, provided there is no indication for atrial pacing, during a 3-year follow-up period [10].

However, to our knowledge, there are still no prospective studies in the literature comparing CRT-DX with two catheters to traditional three-lead (3L) CRT-D.

This study aims to investigate the efficacy and safety of a CRT-DX compared to traditional 3L CRT-D in a single-center prospective study in the long term.

## 2. Materials and Methods

Since 2017 until 1 January 2023, we have consecutively screened CRT recipients: adult patients in sinus rhythm at the time of implantation with a class I or IIa indication for CRT according to the available ESC guidelines and without evidence of sick sinus syndrome (SSS) during titrated beta blocker treatment (resting heart rate > 40 bpm on baseline ECG and maximum heart rate > 85 bpm during the 6 min walk test) were considered suitable either for a DX or a 3L CRTD system. The decision regarding system type was left to the discretion of the electrophysiologist performing the implantation.

Patients with an indication for atrial pacing, upgrade recipients, and permanent AF patients were not considered for the implantation of a DX system. An atrial signal amplitude ≥ 0.6 mV without the DX amplification during respiratory cycles without far-field R-wave oversensing (FFRWO) was the criterion for the DX implantation, whereas an atrial signal amplitude of at least 1 mV without FFRWO was the implanting criterion for atrial leads.

After implantation, CRT setting was optimized in each patient before hospital discharge or at the 2 weeks follow-up visit by echocardiography, ensuring the longest diastolic filling time without A-wave truncation. LV-only pacing was considered in every patient with a PR interval ≤ 240 ms, otherwise biventricular pacing with individualized VV interval was programmed. The lower rate was programmed as 35 bpm in the DX group to avoid VVI pacing at the lower rate, whereas it was programmed as 40 bpm in the 3L group.

Clinical and device-related data were collected at implantation and during follow-up, including echocardiography and device interrogation. Any change in device programming, percentage of biventricular/LV-only pacing, and any arrhythmic events recorded or treated by the device were documented.

The clinical endpoint is a combination of cardiovascular death, HF hospitalization, and new-onset AF. The echocardiographic endpoint was measured by two cut-offs used in the literature: left ventricular end-systolic volume (LVESV) reduction ≥15% or ≥9.3%, as in the most recent and larger CRT trials, respectively [17,18,19].

We also assessed the comparability of the two systems in terms of freedom from lead failure, specifically the durability of atrial sensing in the long term of the DX lead.

Eventually, atrial sensing detected by the two systems and need for atrial pacing during follow-up were evaluated.

The statistical analysis was performed using Stata^®^ software (StataSe 18, StataCorp LLC, College Station, TX, USA). Categorical data are presented as counts and percentages and tested with either χ^2^ tests or Fisher’s exact tests, as appropriate. Continuous variables are expressed as the mean ± standard error, and have been tested with two-tailed Student’s *t*-test. Univariate and then multivariate analysis were conducted if significant differences between the two groups had emerged, in order to test for possible variables interference. Kaplan–Meier survival curves were generated for visual comparison of clinical endpoints and different leads performance. Events during follow-up were analyzed using the Cox proportional hazards model. The chosen level of statistical significance was 0.05.

## 3. Results

### 3.1. Screening and Enrollment

**Figure 1:**
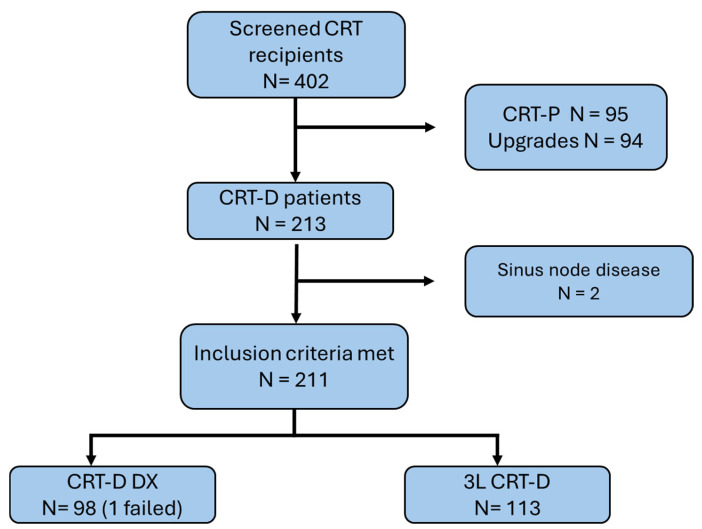
CRT patients screening.

Overall, we screened 402 consecutive CRT recipients: 95 received a CRTP, 94 were upgrades from an existing ICD or pacemaker, and 213 were first-time CRT-D implants. Amongst them, 5/402 (1.2%) had SSS with indication to atrial stimulation and received devices with an atrial lead programmed in the rate responsive mode (2 CRTP recipients, 2CRTD recipients, 1 upgrade patient). Hence, 211/213 met the criteria for implantation of a DX system, which represents 99% of first-time CRTD recipients. Based on the implanter decision, 97 received a DX system and 113 a 3L system. One patient initially enrolled in the CRT-DX group experienced a failure in the implantation of the LV lead due to inadequate coronary venous anatomy and was therefore excluded from the analysis, resulting in a total of 210 patients analyzed Figure 1.

All DX lead implants were achieved successfully owing to atrial sensing, and similarly no atrial lead implant failed because of an inadequate atrial signal. Although no statistically significant differences were observed, a slight trend toward reduced procedural and fluoroscopy times was observed in the DX group (procedural time: 104 ± 3.5 vs. 113 ± 3.7 min, *p* = 0.09; fluoroscopy time: 18 ± 1.6 vs. 21 ± 1.7 min, *p* = 0.21).

All 97 devices implanted in the DX group were manufactured by Biotronik ^®^ (Berlin, Germany), while in the 3L group (*n* = 113), devices were distributed as follows: Medtronic (Dublin, Ireland)^®^ (77), Biotronik^®^ (Berlin, Germany) (25), Boston Scientific^®^ (Marlborough, MA, USA) (6), and St. Jude-Abbott^®^ (Abbott Park, IL, USA) (5). Leads from the same manufacturer as the device were generally implanted, except when an LV active-fixation lead (Medtronic^®^) was employed.

The clinical characteristics of the population at the time of implantation are shown in Table 1, with substantial homogeneity between the two groups, except for a higher rate of LBBB, higher LVESV, and a lower rate of antiarrhythmic therapy in the DX group.

### 3.2. Clinical and Echocardiographic Results

The two groups did not differ significantly in terms of the composite endpoint, evaluated both at one year and at the last available follow-up. At one year, 87% of patients in the DX group and 80% in the 3L group were free from the clinical endpoint (risk ratio, 0.64; 95% CI, 0.33–1.21; *p* = 0.23) (Table 2).

**Figure 2:**
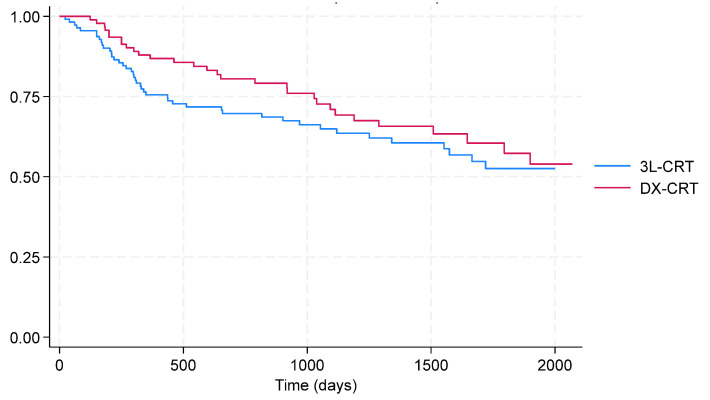
Freedom from composite endpoint Kaplan–Meier curves.

During a mean follow-up of 46.5 ± 1.9 months, 68% of DX patients and 60% of 3L patients remained free from the clinical endpoint. The Kaplan–Meier analysis showed no significant difference between groups (log-rank *p* = 0.23, Figure 2), and in the Cox proportional hazards model, the hazard ratio was 0.76 (95% CI, 0.47–1.20; *p* = 0.23). See Table 2.

The trend toward a lower risk of the composite endpoint in the DX group was no longer evident after adjustment for LBBB, QRS duration, renal disease, and LV-only pacing (adjusted HR = 0.98; 95% CI, 0.58–1.65; *p* = 0.87) (Table 3).

No statistically significant differences were observed between the two groups regarding HF events, new-onset AF, heart transplantation/LVAD implantation, all-cause mortality, or cardiovascular mortality, both at one year and at the end of follow-up.

Based on the echocardiographic criteria of at least 9.3% reduction in LVESV, an overall CRT response rate of 80% at one year and 75% at the end of follow-up was observed. Using the stricter cut-off of −15% LVESV reduction, we observed a 75% response rate at 1 y and 71% at the end of follow-up (Table 4).

Since a higher rate of echocardiographic response (≥9.3% reduction in LVESV) at one year was initially observed in the DX group (88 vs. 75%, *p* = 0.02), we subsequently performed a multivariate analysis, adjusting for the other clinically significant variables that had tested positive at univariate analysis (LBBB, QRS duration, chronic kidney disease-CKD): only the presence of baseline LBBB or CKD retained the ability to distinguish responders from non-responders (Table 5).

### 3.3. Device Data and Leads Performance

Electrical parameters remained stable throughout the follow-up, as shown in Table 6. Notably, atrial sensing values in the DX were significantly higher than in the 3L group both at implantation and during overall follow-up, thanks to the system’s intrinsic signal amplification (DX 5.6 ± 0.4 mV vs. 3-lead 3.3 ± 0.2 mV; *p* < 0.01). In this regard, only four patients in the DX group recorded atrial sensing values below 1 mV, compared to two patients in the 3L. No clinically significant differences were recorded in the electrical parameters of the right and left ventricular leads.

Both groups showed an optimal CRT delivery percentage with preserved AV synchrony (DX 94 ± 1.6% vs. 96 ± 1.5%; *p* = 0.30), and a greater number of LV-only paced patients in the DX (64 ± 5% vs. 50 ± 4.8%, *p* = 0.04). No significant differences in PVC % between groups were recorded (2.5 ± 0.5 vs. 2.2 ± 0.3, *p* = 0.59).

As shown in Table 1, around 88% of patients were on beta blocker treatment at baseline. Mean beta blocker dosage, expressed as metoprolol equivalents (see Supplementary Table S1 for drug dosage conversion), was 85 mg, with a mean increase of 19% by the end of follow-up (DX: 18.9 ± 2.1% vs. 3L: 19.2 ± 2.3%, *p* = 0.7).

Atrial arrhythmia burden was low in both populations (4.5 ± 1.9% vs. 5.2 ± 2.1%; *p* = 0.80), and the atrial pacing percentage in the 3L was 3.6 ± 1.2%.

A total of 31/210 patients (15%) received device therapy for sustained ventricular arrhythmias, 61% of which occurred in the VF zone (HR > 200 bpm). The first arrhythmic event occurred on average 2.8 ± 0.4 years after implantation, with a mean duration of 19.5 ± 2.01 s. The overall ATP efficacy rate was 60% (84% in the VT zone and 25% in the VF zone), with no statistically significant differences between the two groups.

As expected, the rate of VA events was significantly higher in the non-responder population (28.6% vs. 8.8%, *p* = 0.01).

During follow-up, 3 out of 210 patients (one left-sided and two right-sided implants) experienced inappropriate shocks, all caused by inappropriate detection of SVT: all were managed by device reprogramming.

The overall complication rate related to the RV leads requiring system revision was 2.1% in the CRT-DX group and 0.9% in the 3L group at one year (*p* = 0.59), while by the end of follow-up, the lead-related complication rate was 5.2% in the DX group and 1.8% in the 3L group (*p* = 0.25) (Table 7).

In the CRT-DX group, five patients had ventricular oversensing due to insulation defects, two of which proved to be a lead fracture. In two cases, switching of the right and left ventricular leads was possible owing to IS-1 lead connections, an approach previously described by our group for DF-1 devices [20,21]. In one case, repairing the lead insulation was sufficient, while in the remaining two cases, replacement of the damaged lead was necessary.

In the 3L group, one right ventricular lead dislodgement and one lead fracture were observed.

**Figure 3:**
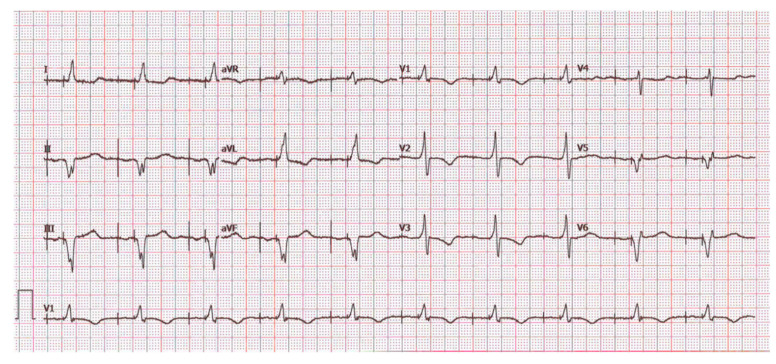
Paced atrial rhythm at the lower right atrium by the floating dipole of the DX lead in 83-year-old patient who developed sinus node disease.

By the end of follow-up, the atrial complication rate was as follows: three atrial lead displacements occurred in the three-lead group; one case of atrial undersensing requiring revision to enable CRT (the dipole had moved up in the SVC due to coiling in the atrium with poor tightening of the sleeve suture); and one case of upgrade to three leads due to onset of sick sinus syndrome in the DX group. The latter patient developed marked sinus bradycardia and chronotropic incompetence (max heart rate 50 bpm) at 83 years, 4 years after implantation despite withdrawal of bisoprolol; thus, the floating atrial dipole was used for atrial pacing at an output of 4 V@0.4 ms (Figure 3). An upgrade to 3L occurred at the time of generator replacement.

## 4. Discussion

Our observation reports enduring performance at long-term follow-up either in terms of clinical response to CRT or lead-related complications when the DX technology is compared to 3L CRT technology in patients without indication to atrial stimulation.

### 4.1. Feasibility and Applicability of the DX Technology in CRT Recipients

Currently, no randomized trials comparing conventional CRT-D and CRT-DX are available in the literature. In a prospective single-center study published by our group in 2017, it was shown for the first time that CRT could be effectively achieved using a DX system, provided there was no indication for atrial pacing [10].

In a retrospective registry Shaik et al. reported similar CRT response and reduction in complications by DX technology compared to 3L system on 240 patients over an average follow-up of about 15 months. Specifically, six patients had atrial lead displacement in the 3L group requiring revision vs. none in the DX group [11].

The recent prospective multicenter observational experience by Kolb et al. clearly reports that the DX technology may be safely proposed to any CRT candidate by a simple screening method to test sinus node competence: a resting heart rate > 40 bpm and an increase in sinus rate above 100 bpm during exercise, detected by any available electrocardiographic means [22]. This method is very similar to ours, for the heart rate during ordinary activity, which is lower in our study (>85 bpm) but requires that maximum tolerated beta blockers or ivabradine, has been achieved. Though follow-up duration is remarkably different across studies, by gathering all the 327 CRT-DX recipients it appears that only 1 patient needed atrial stimulation 4 years after device implantation as an octogenarian [11,22].

### 4.2. Clinical Endpoints

In our study, cardiac resynchronization therapy (CRT) using a two-lead DX system demonstrated effectiveness that was at least similar to that reported in the literature, and comparable to our reference cohort treated with 3L-CRT. Over a mean follow-up of approximately 4 years, no statistically significant differences were observed in terms of clinical events, LV reverse remodeling, and lead failures. The development of sinus node disease with need of atrial stimulation occurred in 1/211 patients.

The composite endpoint of cardiovascular death, heart transplantation or LVAD implantation, HF events, and/or new-onset AF occurred in 16% of patients at one year and 36% at approximately four years. These results—along with a 2.5% annual cardiovascular mortality rate—are consistent with the most recent and largest randomized CRT trial, which reported an annual cardiovascular mortality of 3% [23].

### 4.3. CRT Delivery

In our population, the percentage of CRT delivery (94 ± 1.6% vs. 96 ± 1.5%; *p* = 0.3) and PVC percentage (2.5 ± 0.5 vs. 2.2 ± 0.3, *p* = 0.59) did not show statistically significant differences between systems, and only one patient required a system revision due to undersensing over a long follow-up. The high CRT percentage together with the low PVC percentage, with no significant differences between the two groups, confirms that AV synchrony was 100%, as shown by device counters. This finding is consistent with our previous pioneering experience, since an increase in the PVC counter can indicate P-wave undersensing and any potential underperformance of atrial sensing with the DX system would reduce CRT delivery [10]. Similarly, other observational studies have reported extents of CRT delivery with DX systems comparable to 3L-CRT [10,11].

### 4.4. Ecocardiographic Response

The high CRT delivery rate corresponded to 80% mean echocardiographic response. Left ventricular end-systolic volume decreased by an average of 47 mL (−58 vs. −36 mL; *p* = 0.04) and left ventricular ejection fraction increased by 11.6% on average (12.7% vs. 10.4%; *p* = 0.3). These response rates are notably higher than typically reported in the literature, where non-response rates range from 30% to 50% [24].

In 2020, Shaik et al. published a sub-analysis showing that the CRT-DX system offers a similar reverse remodeling response compared to 3L-CRT similarly to our observation, with a lower lead-related complication rate [11]. Similarly, the recent report of Kolb et al. shows a very high quality CRT with DX technology [22].

These results highlight the importance of patient selection, accurate identification of the venous target with the greatest electro-mechanical delay for LV leads positioning (as evidenced by optimal intraoperative Q-LV and RV-LV timing measurements), and post-implant optimization process by means of AV and VV delays tailoring.

The most recent data acquired on CRT over the years, together with technological advancements in LV lead performance that sustain CRT efficacy during follow-up, compel us to reconsider CRT expected response and establish new benchmarks for the comparison with alternative resynchronization strategies (i.e., conduction system pacing).

### 4.5. Arrhythmic Events

The low atrial pacing rate in the 3L group (3.6 ± 1.2% vs. 0% in the CRT-DX group) was associated with a very low AF burden (4.5 ± 1.9 vs. 5.2 ± 2.1; *p* = 0.8), aligning with the hypothesis that reduced atrial pacing may help prevent AF onset [25,26,27].

The long-term ventricular arrhythmic event rate is consistent with the most recent data reported in the literature [28]; notably, we observed in both groups a very low incidence of inappropriate shocks (1.4%), largely inferior to the results of most large trials on transvenous and subcutaneous ICDs (ranging 7–13%) which can be at least be partially attributed to the routine programming strategy adopted at our center, characterized by high-rate VF cut-offs and prolonged detection [5,14,29,30].

This also aligns with findings from several studies supporting the DX lead’s performance in terms of atrial sensing quality [14,15,31,32].

### 4.6. Leads’ Performance

Overall, no significant differences were observed in terms of right lead complications (DX vs. conventional) or of atrial sensing performance (DX sensing dipole vs. standard atrial lead).

We did not observe a higher rate of complications related to Linox ICD leads compared to later models (2/5), as it had previously been raised by Oosterwerff et al. [33].

The development of SSS in the CRT population was a rare event, with only one upgrade involving the atrial lead, which was performed at the time of generator replacement. On the contrary, there were three system revisions due to atrial lead dislodgement in the 3L group (1.8%).

The complication rate associated with CRT implantation varies widely across studies, ranging from approximately 3% to 11%, primarily due to lead revisions—particularly of the left ventricular lead [34,35,36]—as well as a higher risk of infection compared to PM/ICD implantation. In fact, lead burden is now included in major risk scores for predicting implant-related infections [37].

This variability is attributable to several factors, foremost among them the operator’s experience and the implanting center’s volume, but also patient-related factors such as sex, comorbidities, presence of advanced HF, or a high burden of AF [35].

In the 3L, three cases of symptomatic left subclavian vein thrombosis (associated with arm edema) were reported, necessitating prolonged oral anticoagulation (no cases occurred in the DX group; *p* = 0.25).

Though the number is small, this finding could support the rationale for minimizing the number of implanted leads in order to reduce venous crowding, which may underlie such complications. It is well-established that the presence of three or more leads in a thoracic venous axis increases the risk of venous occlusion eightfold [38]. Furthermore, three or more leads double the risk of clinical failure of lead extraction due to infection and/or system malfunction, as shown in the ELECTRa European registry [29].

### 4.7. Limitations

This study is a prospective non-randomized single-center study. The potential confounding factors related to partial heterogeneity in the population were attenuated performing univariate and then multivariate analysis if significant differences between the two groups had emerged. Our observations should be confirmed also by randomized clinical trials, but the population numerosity and the long follow-up add importance to the results we observed.

## 5. Conclusions

In real-world CRT practice, atrial pacing indication is present in fewer than 2% of patients, making DX technology suitable for the vast majority of CRT recipients. Our single-center study shows that the CRT-DX system enables clinical and instrumental CRT response in the long term, and complication rates were comparable to 3L CRT-D systems in patients without a need for atrial pacing. This may confer a long-term advantage due to reduced intravascular hardware.

## Figures and Tables

**Table 1:** Patients’ characteristics at baseline.

	All (*n* = 211)	DX (*n* = 98)	3L (*n* = 113)	*p* Value
Age (years)	66.4 ± 1.5	65.5 ± 1.1	67.2 ± 1.1	0.28
Female %	30.3	29.5	30.9	0.82
BMI (Kg/m^2^)	27.0 ± 0.3	27.3 ± 0.5	26.7 ± 0.4	0.37
NYHA class I-II, *n* (%)	138 (65%)	68 (69%)	70 (62%)	0.25
Underlying HD *n* (%)				
Ischemic post-MI HD	43 (20)	21 (21)	22 (19)	0.69
Ischemic HD	12 (6)	3 (3)	9 (8)	0.15
Hypertrophic HD	11 (5)	4 (4)	7 (6)	0.55
Valvular HD	16 (8)	8 (8)	8 (7)	0.75
Dilated CMP	112 (53)	53 (54)	59 (52)	0.73
Other *	14 (7)	8 (8)	6 (5)	0.40
Diabetes *n* (%)	61 (29)	29 (30)	32 (28)	0.83
Insulin-dependent	19 (9)	10 (10)	9 (8)	0.57
Creatinine (mg/dL)	1.18 ± 0.5	1.19 ± 0.7	1.18 ± 0.4	0.87
COPD, %	28 (13)	13 (13)	15 (13)	0.99
Stroke/TIA/PAD *n* (%)	15 (7)	4 (4)	11 (10)	0.11
History of AF, *n* (%)	51 (24)	24 (25)	27 (24)	0.92
History of AVB, *n* (%)				
I° AVB	45 (21)	16 (16)	29 (26)	0.09
II° AVB	11 (5)	4 (4)	7 (6)	0.48
III° AVB	3 (1)	1 (1)	2 (2)	1
HR (bpm)	65 ± 13	66 ± 15	65 ± 12	0.37
QRS duration (ms)	159 ± 17	160 ± 18	159 ± 17	0.75
LBBB	143 (68)	75 (77)	68 (60)	0.01
RBBB	6 (3)	3 (3)	3 (3)	0.84
LAFB	16 (8)	4 (4)	12 (11)	0.08
RBBB + LAFB	16 (8)	5 (5)	11 (10)	0.22
IVCD	33 (16)	11 (11)	22 (19)	0.11
EF (%)	30 ± 6	29 ± 6	30 ± 6	0.37
LVESV (mL)	140 ± 3.4	149 ± 5.5	133 ± 4.1	0.02
LVESV(i) (mL/m^2^)	73 ± 1.8	77.1 ± 2.8	68.5 ± 2.3	0.04
Medical therapy (*n*, %)				
Beta blockers	186 (88)	87 (89)	99 (88)	0.76
ARNI, ARB, ACEi	183 (87)	85 (88)	98 (88)	0.98
SGLT2i	46 (22)	25 (26)	21 (19)	0.22
MRA	133 (63)	64 (66)	69 (62)	0.51
Loop diuretic	166 (79)	75 (77)	91 (81)	0.48
Antiarrhythmic drugs	38 (18)	12 (12)	26 (23)	0.04

Continuous variables are expressed as mean ± mean standard error. * Chemotherapy and/or radiotherapy, infiltrative diseases (amyloidosis, Fabry disease), GUCH (Grown Up Congenital Heart). BMI: body mass index, HD: heart disease, CMP: cardiomyopathy COPD: chronic obstructive pulmonary disease, TIA: transient ischemic attack, PAD: peripheral artery disease, AF: atrial fibrillation, AVB: atrioventricular block, HR: heart rate, LBBB: left bundle branch block, RBBB right bundle branch block, LAFB: left anterior fascicular block, IVCD: intraventricular conduction delay, EF: ejection fraction, LVESV(i): left ventricular end-systolic volume (indexed); ARNI: Angiotensin Receptor–Neprilysin Inhibitor, ARB: angiotensin receptor blocker, ACEi: angiotensin converting enzyme inhibitor, MRA: mineralocorticoid receptor inhibitor.

**Table 2:** Clinical follow-up.

	All	DX	3L	*p* Value
Primary clinical endpoint at 1 year (%)	34 (16.1)	13 (13.4)	23 (20.3)	0.23
Primary clinical endpoint last follow-up (%)	76 (36.1)	31 (31.9)	45 (39.8)	0.24
HF events (%) after 1 year	20 (9.5)	8 (8.2)	12 (10.6)	0.62
HF events (%) last follow-up	22 (10.5)	10 (1.3)	12 (10.6)	0.95
New AF (%) after 1 year	12 (5.7)	5 (5.2)	7 (6.2)	0.79
New AF (%) at last follow-up	25 (11.9)	14 (14.4)	11 (9.7)	0.28
Heart Tx/LVAD ^1^ (%) at 1 year	4 (1.9)	0 (0)	4 (3.5)	0.13
Heart Tx/LVAD (%) at last fup	10 (4.8)	3 (3.1)	7 (6.2)	0.35
Death for all causes (%) at 1 year	8 (3.8)	2 (2.1)	6 (5.3)	0.30
Death for all causes (%) at last fup	35 (16.7)	14 (14.4)	21 (18.6)	0.45
CV death (%) at 1 year	5 (2.4)	1 (1)	4 (3.5)	0.99
CV death (%) at last follow-up	19 (9)	8 (8.2)	11 (9.7)	0.73
Follow-up duration, months	46.5 ± 1.9	49.6 ± 3.2	43.9 ± 2.2	0.14

^1^ Heart transplant/Left Ventricular Assist Device.

**Table 3:** Multivariate Cox proportional hazards model.

Composite Endpoint at Final Follow-Up	HR	95% CI	*p* Value
DX group	0.98	0.58–1.65	0.93
LBBB	0.64	0.35–1.15	0.14
QRS duration	1.00	0.98–1.01	0.73
CKD	2.52	1.56–4.09	<0.001
LV-only pacing	0.96	0.54–1.68	0.88

HR = Hazard ratio; CI = confidence interval. LBBB: Left bundle branch block. CKD: Chronic kidney disease.

**Table 4:** Echocardiographic response.

	All	DX	3L	*p* Value
Echocardiographic responders 1 year FUP				
- <9.3% LVESV criteria	80%	88%	75%	0.02
- <15% LVESV criteria	75%	81%	70%	0.08
Echocardiographic responders Last FUP				
- <9.3% LVESV criteria	75%	78%	71%	0.39
- <15% LVESV criteria	71%	75%	67%	0.42
LVEDV mean variation 1 year FUP (mL)	−38.1 ± 4	−53.2 ± 6.2	−27 ± 5	0.01
LVEDV mean variation last FUP (mL)	−46.1 ± 6.2	−58.7 ± 9	−33.7 ± 8.2	0.04
LVESV mean variation 1 year FUP (mL)	−44 ± 3.6	−62.4 ± 6.2	−31 ± 4.2	0.001
LVESV mean variation last FUP (mL)	−47.4 ± 5.6	−58.7 ± 8.4	−36.2 ± 7.4	0.04
LVEF mean variation 1 year FUP (%)	11.4 ± 0.74	14.1 ± 1	9.4 ± 1	0.001
LVEF mean variation last FUP (%)	11.57 ± 1	12.7 ± 1.6	10.4 ± 1.5	0.30

Values expressed as % for parametrical variables and mean ± standard error for continuous variables. LVESV: left ventricular end-systolic volume, LVEDV: left ventricular end diastolic volume, LVEF: left ventricular ejection fraction.

**Table 5:** Multivariate logistic regression.

Echocardiographic Response at 1 Year (−9.3% LVESV)	OR	95% CI	*p* Value
DX group	2.06	0.75–5.67	0.16
LBBB	5.77	2.16–15.4	<0.01
QRS duration	1.00	0.98–1.03	0.75
CKD	0.32	0.13–0.78	0.01
LV-only pacing	1.40	0.51–3.81	0.88

OR = Odds ratio; CI = confidence interval. LBBB: Left bundle branch block. CKD: Chronic kidney disease.

**Table 6:** Device parameters at baseline and follow-up.

	Baseline			Follow-up		
	DX	3L	*p* Value	DX	3L	*p* Value
A sensing (mV)	6.3 ± 0.5	3.2 ± 0.52	<0.01	5.6 ± 0.4	3.3 ± 0.2	<0.01
RV sensing (mV)	16.8 ± 0.5	13.2 ± 0.65	<0.01	15.5 ± 0.6	12.4 ± 0.6	0.03
RV impedence (ohm)	586 ± 13	543 ± 12	0.02	535 ± 13	453 ± 10	0.01
RV threshold (V@0.4 ms)	0.6 ± 0.3	0.8 ± 0.2	0.34	0.7 ± 0.1	0.8 ± 0.1	0.20
LV impedence (ohm)	611 ± 18	678 ± 23	0.03	625 ± 19	608 ± 16	0.50
LV threshold (V@0.4 ms)	1 ± 0.7	1 ± 0.5	0.69	1 ± 0.5	1.1 ± 0.5	0.18
Q-LV (ms)	123 ± 4.6	130 ± 6.4	0.32	-	-	
RV-LV (ms)	97 ± 5.1	99 ± 5.6	0.88	-	-	
Tot. CRT delivered (%)				94 ± 1.6	96 ± 1.5	0.30
Biventricular				36 ± 5.1	47 ± 4.8	0.10
LV-only				64 ± 5	50 ± 4.8	0.04
AF burden (%)				4.5 ± 1.9	5.2 ± 2.1	0.8
Arrhyth. events *n* (%)				4 (4.5)	5 (4.8)	0.93
PVC counter (%)				2.5 ± 0.5	2.2 ± 0.3	0.59
A pacing (%)				0	3.6 ± 1.2	

Continuous variables are expressed as mean ± standard error. A: Atrial. RV: Right ventricular. LV: Left ventricular. Q-LV: QRS-LV sensing time interval. RV: RV-LV sensing interval. AF: Atrial fibrillation.

**Table 7:** Device and lead complications.

	All	DX	3L	*p* Value
Device/lead infections	3 (1.4)	1 (1)	2 (1.8)	0.56
Lead failures:				
- Atrial lead/upgrade *n* (%) 1 year	2 (1)	0	2 (1.8)	0.50
- Atrial lead/upgrade *n* (%) last fup	5 (2.4)	2 (2.1)	3 (2.7)	0.99
- RV lead *n* (%) 1 year	3 (1.4)	2 (2.1)	1 (0.9)	0.33
- RV lead *n* (%) last fup	7 (3.3)	5 (5.2)	2 (1.8)	0.25
- LV lead *n* (%) 1 year	5 (2.4)	4 (4.1)	1 (0.9)	0.18
- LV lead *n* (%) last fup	9 (4.3)	7 (7.2)	2 (1.8)	0.08

RV: Right ventricular. LV: Left ventricular.

## Data Availability

The data presented in this study are available on request from the corresponding author due to privacy restrictions.

## Supplementary Materials

The following supporting information can be downloaded at https://www.mdpi.com/article/10.3390/jcm14248746/s1, Table S1: beta blockers equivalent dosages.

## Abbreviations

The following abbreviations are used in this manuscript:

3L	Traditional three leads CRT
A	Atrial
ACEi	Angiotensin converting enzyme inhibitor
AF	Atrial fibrillation
ARB	Angiotensin receptor blocker
ARNI	Angiotensin Receptor–Neprilysin Inhibitor
AVB	Atrioventricular block
BMI	Body mass index
CKD	Chronic kidney disease
CMP	Cardiomyopathy
COPD	Chronic obstructive pulmonary disease
CRT (-D)	Cardiac Resynchronization Therapy (with defibrillator)
CI	Confidence interval
DX	Two Leads CRT with DX system
EF	Ejection fraction
FFRWO	Far-field-R-wave oversensing
GUCH	Grown Up Congenital Heart
HD	Heart disease
HF	Heart failure
HFrEF	HF with reduced ejection fraction
HR	Heart rate/Hazard ratio
IVCD	Intraventricular conduction delay
LAFB	Left anterior fascicular block
LBBB	Left bundle branch block
LV	Left ventricle
LVAD	Left Ventricular Assist Device
LVEDV	Left ventricular end diastolic volume
LVEF	Left ventricular ejection fraction
LVESV	Left ventricular end-systolic volume
LVESV(i)	Left ventricular end-systolic volume (indexed)
MRA	Mineralocorticoid receptor inhibitors
OR	Odds ratio
PAD	Peripheral artery disease
Q-LV	QRS–LV sensing time interval
RBBB	Right bundle branch block
RV	Right ventricle
RV-LV	Right ventricle–Left ventricle sensing interval
SSS	Sick sinus syndrome
SVT	Supraventricular tachycardia
TIA	Transient ischemic attack
Tx	Transplant
