# Supplementary material for: Comparative Analysis of Two-Lead DX-Based CRT Versus Conventional Three-Lead CRT-D: Results from a Single-Center Prospective Study
Source: J Clin Med. 2025 Dec 10;14(24):8746. doi: 10.3390/jcm14248746 (PMC12733999; doi:10.3390/jcm14248746)
Supplement: Supplementary file 1 [file jcm-14-08746-s001.zip › jcm-3999989-supplementary.pdf]

**Metoprolol**	12.5 mg bid	25 mg bid	50 mg bid	100 mg bid
**Carvedilol**	3.125 mg bid	6.25 mg bid	12.5 mg bid	25 mg bid
**Bisoprolol**	1.25 mg qd	2.5 mg qd	5 mg qd	10 mg qd
**Nebivolol**	1.25 mg qd	2.5 mg qd	5 mg qd	10 mg qd

Table S1*: beta blockers equivalent dosages*
